# Initial Results of a Prospective Study and Identification of New Strategies to Increase Traceability of Plasma-derived Medicines

**Published:** 2018

**Authors:** Sheyda Najafi, Ali Vasheghani Farahani, Hedieh Keshavarz-Bahaghighat

**Affiliations:** a *Tehran University of Medical Sciences, Faculty of Pharmacy. *; b *Shahid Beheshti University of Medical Sciences, Faculty of Pharmacy, Department of Pharmacoeconomics and Pharmaceutical Management.*

**Keywords:** Plasma-derived medicines, Traceability, Medical records, Guideline, Documentation

## Abstract

Plasma medicine is an innovative and emerging field used in a broad range of medical conditions. The present study focused on consumption and documentation pattern of plasma-derived medicines in a teaching hospital. A two-step study was conducted from October to December 2015. During the first phase, the patient records receiving plasma-derived medicines including Coagulation Factor VIII, IX, Prothrombin Complex Concentrate, Factor VIII/Von Wilberand Complex, Anti-Hepatitis B Immunoglobulin, Intravenous Immunoglobulin, Anti-Tetanus Immunoglobulin, and Albumin were checked to assess recording details of these medications at the time of administration. Adverse events reported with the mentioned products were examined from traceability viewpoint. The second step concentrated on practical strategies to improve documentation status of plasma-derived medicines in the hospital. We proposed national guideline as the first strategy and a new barcoding system to track and identify drug information of plasma medicines. Of the expected drug information, only generic name, dosage from, and strength were recorded after administration. Post-marketing safety surveillance of the plasma products was poor similarly. Unavailability of suitable instructions was the main reason for documentation deficiency. A guideline was designed and implemented to inform healthcare professionals about essentials of appropriate documentation for plasma-derived medicines. Updated results of the ongoing phase will be submitted soon. Our survey highlights the importance of documentation as a key component of plasma-derived medicines surveillance within the hospitals.

## Introduction

Plasma-derived medicinal products (PDMPs) are manufactured from human plasma and consist of components such as albumin, coagulation factors, and immunoglobulins, which are life-saving therapeutics for a number of chronic and acute diseases (1). A wide variety of plasma proteins have been made available over recent years due to improvements in protein purification technology. The importance of these medicines in treatment of life-threatening diseases is reflected by the fact that World Health Organization (WHO) has included PDMPs in the WHO list of essential medicines (2). Today, PDMPs are thought to be very safe, but that was not always the case. The beginning of a major transformation was the recognition of extensive transmission of human immunodeficiency virus (HIV) and hepatitis C in mid-1980 when these viruses in plasma supply of infected donors, contaminated thousands of the hemophilia community (3). Infectious non-enveloped viruses also were found in certain PDMPs during the 1990’s and early 2000’s. Moreover, several cases of Creutzfeldt-Jakob disease (CJD) infection by blood transfusion in the UK showed strong evidence that CJD may also be transmitted through blood transfusion (4-7). The transmissibility of prion diseases like scrapies in sheep, bovine spongiform encephalopathy in cattle, and CJD in humans are new concerns (8). Additional blood-borne pathogens include Treponema pallidum, human parvovirus B19, and more barely hepatitis A (8-10). Recently, the published reports on frequency of viremic blood donations and studies on plasma pools reveal that plasma pools used for manufacture of medicinal products can be contaminated with Hepatitis E virus (11).

The preparation of PDMPs is based on precise safety measures, including screening of blood donors, rigorous plasma testing for infectious agents, and pathogen inactivation procedures (12). Providing safe and effective medicine from blood donation to administration of a PDMP is a prolonged and complex process, with multiple checkpoints. Safe products are the result of progress in donor screening methods, laboratory tests to detect blood-borne viruses, quality control analysis, viral inactivation, and manufacturing processes. The risk of HIV, hepatitis B, and C virus transmission have been approximately eliminated by these improvements (3, 13-15).

Regulation of PDMPs is the responsibility of national medicines regulatory authorities. Over the past few decades, these authorities have dealt with serious and complex challenges at a scientific, technological, and regulatory level to ensure that these biological products possess high standards of safety and efficacy (3, 15, 16). Countries should set up a national system for post-marketing surveillance of PDMPs. National regulatory authorities should consider a system for enforcing the recall of batches, invalidating approvals, and notifying manufacturers, users, and the medicines regulatory authorities of any importing countries about such decisions (3, 17). Information on the collection and control of the starting plasma should be documented as part of the licensing procedure. This system aims to ensure quality and traceability of each plasma unit from the donor, through the manufacturing process to the recipient of the product and vice-versa (3, 17). In Iran, PDMPs as a subset of biological products are under regulation of biologics office of Iranian Food and Drug Administration (IR-FDA) recognized as fully functional in May 2010 by the WHO. Registration, lot release, GMP inspection either for plasma collection centers and fractionators, clinical trial and pharmacovigilance affairs are the main responsibilities of the biologics office. 

Documentation is vital to guarantee traceability of PDMPs. Traceability is required and taken to quickly retrieve history, use, and localization of a PDMP at each step of the supply chain: from the blood donation, through the production, distribution, and dispensing up to the administration (18). Retrieval becomes possible from a PDMP batch number of the blood donation that were pooled for this batch preparation and the recipients of this batch. Traceability must be maintained throughout this chain (19). It is powerfully recommended that every time a PDMP is administered to a patient, the brand name and batch number of the product are recorded in order to provide a link between the patient and the batch of the product according to the Note for Guidance on the warning on transmissible agents in the Summary of Product Characteristics and Package Leaflets for PDMPs. This is to make sure that the Marketing Authorization Holder for this product or a manufacturer, using a batch of a PDMP, and the Competent Authorities would be informed in exceptional circumstances (4).

The aim of the study was to enhance traceability of PDMPs based on a preliminary observational study.

## Methods

A primary study was carried out to evaluate documentation and traceability of PDMPs. Due to necessity of complete recording of drug information in patient files, we designed an observational study to explore documentation status of PDMPs in a teaching hospital. The total number of PDMPs consumed during the study period included Coagulation Factor VIII, IX, Factor VIII/Von Wilberand Complex, Prothrombin Complex Concentrate, Intravenous Immunoglobulin, Albumin, Anti-Tetanus Immunoglobulin, Anti-Hepatitis B Immunoglobulin. Recombinant type of coagulation factors XIII and VII were excluded from this survey. Using the Morgan Table, the required sample size was determined and the patients were selected randomly. Patient information was extracted from inpatients hospital IT system in a daily schedule within three months of October to December 2015 (20).

Among PDMPs, coagulation factor VIII and Anti-Tetanus immune globulin were extensively used in the hospital. With regard to other products with lower consumption rate, all patients receiving the PDMPs were included. Nursing reports of drug administration for PDMPs were investigated by a pharmacist. These records were expected to include product brand name, dosage form and strength, and batch number. In addition, those wards with the higher consumption rates were determined in the hospital.

**Table 1:** Sample size of the studied PDMPs.

**PDMP**	**Sample size**
Coagulation factor VIII	132
Coagulation factor IX	74
Albumin	169
IVIG	67
Factor VIII/Von Wilberandcomplex	25
Anti-Tetanus immune globulin	155
Anti-hepatitis B Immune globulin	18
Prothrombin complex concentrate	25

**Table 2:** The consumption pattern of PDMPs in the hospital wards

**PDMP**	**Ward**
Anti-Tetanus Immunoglobulin	Emergency department
IVIG	Neurology, kidney transplant, and Neonatal Intensive Care Unit respectively
Anti-hepatitis B immunoglobulin	Liver Transplant Intensive Care Unit and Hepatobiliary Department respectively
Coagulation factors (VIII and IX)	Haemophilia Clinic
Prothrombin Complex Concentrate,	Haemophilia Clinic
factor VIII/Von Wilberandcomplex	Haemophilia Clinic
Albumin	Intensive Care Unit, Nephrology, and Liver Transplant Unit respectively

The sample size of the studied PDMPs has been illustrated in Table 1. Adverse drug events reported with PDMPs to Adverse Drug Reaction Registry were investigated from traceability view point in the study period. The yellow cards were expected to include complete information of the suspected medicine and manufacturer (brand name and batch number). As a secondary outcome, nursing feedback regarding to barriers to good documentation was sought by a questionnaire. The information was gathered in data collection forms designed by the pharmacist and analyzed subsequently.

According to the findings of the first phase of study, two strategies were proposed including (1) guideline enforcement to inform healthcare professionals about significance of recording and (2) development of a new tracking method to facilitate the surveillance of PDMPs after administration. In other words, the second phase of study started to examine the efficacy of these methods to overcome the poor documentation in the hospitals.

A national guideline clarifying the importance of documentation for PDMPs was designed and implemented by IR-FDA on September 2016. According to the guideline, nurses in all hospitals should record batch number and brand name of plasma-derived medicines in nursing administration sheet at the time of administration. The guideline was also added to Iran National Accreditation standards for hospitals and the deputy hospital should monitor the execution. The second strategy uses barcode scanning equipment to scan a barcode on the label of PDMPs. This system is under further investigation and aims to facilitate the tracking of PDMP information.

## Results

We report the initial results of the study concerning traceability of PDMPs in the hospital. The distribution of PDMP based on consumption in the medical wards has been illustrated in the Table 2.

Our assessment revealed poor documentation for PDMPs in patient files. All the studied files lacked manufacturer information including batch number and brand name. The only recorded information included dosage form, generic name, and strength of the medication. 

There were three main reasons for poor documentation among nurses including lack of awareness about necessity of documentation, lack of guidelines informing healthcare professionals, and finally nursing workload respectively. 

In this study, in the case of occurrence of an adverse event or any drug-related problem, the suspected drug brand name and batch number was just traceable within first hours of the event. Generally the drug package was thrown promptly after the administration but by checking the drug stock in ward and pharmacy resource, the batch number might be achieved. In three (out of four) reports of adverse events in the study time period, due to high consumption in the medical ward or multiplicity of the product batch numbers in the hospital inpatient pharmacy, the suspected batch number could not be detected. Only one report included complete information of the administered plasma product which was recorded promptly after the administration.

With regard to the importance of documentation and some cases of adverse events which should be traced as soon as possible, a national guideline was designed and enforced by IR-FDA. To facilitate the documenting process, a new barcoding system was also proposed by IR-FDA to identify and track drug information. We are now in the data collection step after guideline implementation. Regarding to barcoding system, once the design of all companies finishes, our second phase of data collection will begin. To analysis the effectiveness and level of significance of these new instructions, the second phase of study is under precise investigation. 

## Discussion

Our referral hospital with a total number of 1400 beds is the largest hospital in Iran which admits a high number of patients annually. In addition, the majority of plasma products are prescribed and utilized in this referral hospital. More than half of PDMPs are manufactured from voluntary blood donations (21). The transmission of blood-borne pathogens including bacteria, viruses, and prions is of particular concern in the preparation of PDMPs (8). While human plasma is a precious source of many therapeutic proteins, insufficiently screened human plasma or plasma components can transmit a variety of pathogens, such as HIV, hepatitis B, hepatitis C, and West Nile virus (9, 22). This can raise more questions about the safety of PDMPs. 

The manufacturing of PDMPs is subject to standard safety measures to prevent infections resulting from the use of the medicine (21). These measures consist of accurate selection of donors, screening of individual donations and plasma pools for specific markers of infection, and the inclusion of effective manufacturing steps for the inactivation/removal of viruses (6, 23). The present measures taken are considered sufficient for enveloped viruses such as HIV, hepatitis B, and C virus. (24) However, within administration of these medicines, the risk of transmitting infective agents cannot be entirely excluded. This also applies to unexplored or emerging viruses and other pathogens. Even adequately screened plasma is always suspected to carry a very low risk for the transmission of pathogens, since screening tests presently available cannot rule out all potential pathogens nor completely anticipate future blood transfusion transmitted agents (9).

A blood-borne disease can be potentially transmitted to a large number of recipients by a batch of starting plasma containing a single contaminated unit of plasma. Multiple intermediate products and consequently various batches of the final product can be manufactured from the contaminated plasma pool. Therefore, the preventive measures aim to minimize the contamination of the starting material and are responsible for the safety of PDMPs (25). Recognizing the clear evidence for contamination of plasma donations and pools, manufacturers are encouraged to perform primary risk evaluations for their PDMPs on the basis of the available information.

Following the identification of the first 164 cases of HIV among 2734 hemophilia patients in Iran, IR-FDA set up precautionary actions to lower the possibility of pathogen transmission by PDMPs and kept under careful surveillance to ensure the relative safety and efficacy of these medicines. Risk analysis is crucial for evaluating the safety of PDMPs (26, 27).

In this study, all explored records lacked the expected data consisting of the manufacturer batch number and brand name. Since possible safety problems may be batch-related, a strong recommendation to health professional is that, every time that a PDMPs is administered to a patient, the brand name and batch number of the product should be documented in order to provide a link between the patient and the batch of the product (24).

According to the guideline for reporting of adverse drug events, the manufacturer information (brand name and batch number) is essential elements of yellow cards to report adverse events. So, it is suggested to inform healthcare professionals about necessity of documentation by guideline implementation. Healthcare professionals should be encouraged to report unexpected adverse events occurring after administration of PDMPs to the manufacturers and national regulatory authorities (2, 3, 28).

This is the first study in Iran to examine the documentation deficiencies of the PDMPs. The follow-up step will evaluate the efficacy of guideline enforcement and implementation of new barcoding system in all hospitals. Data collection for the second phase of study is open-ended because confirmation of the efficiency of such interventions requires more time.
